# Topiramate for hypoxic ischemic encephalopathy: A systematic review protocol

**DOI:** 10.1097/MD.0000000000018704

**Published:** 2020-04-24

**Authors:** Guoming Chen, Yijun Chen, Yaying Xie, Ruilan Huang, Tengyu Chen, Peiyu Shi, Zhaoping Zhang, Yingyue Hou, Wanli Xing, Li Wei

**Affiliations:** a Guangzhou University of Chinese Medicine; b First Affiliated Hospital of Guangzhou Medical University; c National Clinical Research Center for Respiratory Disease; d Department of Pharmacy, First Affiliated Hospital of Guangzhou Medical University, Guangzhou, China.

**Keywords:** hypoxic ischemic encephalopathy, protocol, systematic review, topiramate

## Abstract

**Background:**

Hypoxic ischemic encephalopathy (HIE) is brain injury caused by different reasons and the most common diagnosed is neonatal HIE. Most of the existing treatments have their own shortcomings or there are still some unexplained mechanisms in it. Topiramate (TPM) is a new drug for the treatment for seizures in neonates with HIE, but is currently used off-label. Our protocol aims to access the efficiency and safety of TPM for HIE.

**Methods and analysis:**

Eight databases will be searched by 2 independent researchers for the article on the topic of using TPM as treatment for HIE, including PubMed, the Cochrane Central Register of Controlled Trials (Cochrane Library), Embase, and Web of Science, China National Knowledge Infrastructure (CNKI), Chinese Biomedical Literature Database (CBM), Wang Fang Database and Chinese Science and Technology Periodical database (VIP). The included papers are those published from the established date of the databases to 2019. The therapeutic effects based on the grade of neonatal behavioral neurological assessment will be regarded as the primary outcomes. RevMan V5.3 will be used to compute the data synthesis and carry out meta-analysis. The risk of bias will be appraised by the Cochrane risk of bias tool. Rare ratio for dichotomous outcomes and mean different for continuous data will be expressed with 95% confidence intervals (CI) for analysis. A random effects model or a fixed effects model will be employed, when heterogeneity is found or not. Subgroup analysis and sensitivity analysis will be applied if the heterogeneity is obvious.

**Results:**

This study will provide the recent evidence of TPM for HIE from reducing seizure acticity.

**Conclusion:**

The conclusion of this study will provide proof to evaluate if TPM is effective and safe in the treatment of HIE.

PROSPERO registration number: PROSPERO CRD42018117981

## 1 Introduction

Hypoxic ischemic encephalopathy (HIE) is brain injury caused by different reasons and the most common diagnosed is neonatal hypoxicc ischemic encephalopathy.^[1,2]^ Neonatal HIE is brain injury caused by lack of perfusion and oxygen deficit which usually happens because of umbilical cord compression or placental abruption.^[3]^ Besides, Non-neonatal HIE is found in various causes of severe cerebral tissue ischemia and hypoxia, commonly led by respiratory and cardiac arrest, trauma, severe hypotension or hypertension or venous sinus thrombosis,^[4,5]^ but also can be seen in shock, CO poisoning, epileptic status, myasthenia gravis and so on. The diagnosis of HIE in developed country occurs between 1 and 2/1000 live births, while in developing countries it may be increase to 26/1000 live births.^[6]^ Furthermore, neonatal HIE is one of the main causes of death among newborns.^[7]^ Newborns with HIE have prominent mortality and morbidity, according to the data provided by the existing study, about 20% of cerebral palsy in childhood are caused by HIE,^[8]^ and the incidence rate of neonates with seizures increase from 30% to 90%.^[9]^ The current effective treatments for HIE include Hypothermia, Melatonin, Allopurinol, Magnesium sulfate (MgSO4), Erythropoietin (EPO), N-acetylcysteine (NAC), Xenon and so on.^[10]^ Despite the interventions are various, most of them have their own shortcomings or there are still some unexplained mechanisms in it. For example, the effects of cooling therapy for HIE in middle-income countries without basic newborn care is difficult to achieve the desired effect.^[11]^ Therefore, development of new drugs is very significant and urgent for curing HIE.

Topiramate (TPM) is an anti-epileptic widely used in different groups of people.^[12]^ It is a new type anti-epileptic drug which has the potential neuroprotective effect.^[13]^ It is mainly used as an adjuvant drug for other anti-epileptic drugs and is used for different kinds of partial and generalized seizures.^[13]^ The anti-epileptic mechanisms of TPM are listed below:

(1) selective blocking voltage dependent sodium channel;
(2) acting on gamma-aminobutyric acid receptors, enhance the neuroinhibitory effect of gamma-aminobutyric acid;
(3) acting on glutamate receptors, antagonizes the glutamate receptors of Marine acid / AMPA, and reducing the excitatory effect of glutamate mediated nerve.^[14,15]^

TPM has good long lasting curative effect and has no obvious tolerance. This potential reducing neural excitatory effect manifests that TPM can be used as a candidate drug for hypoxic ischemic brain injury.^[16]^ Research with animal models such as rats and piglets have proved that TPM used alone can reduce the brain injury in neonatal HIE.^[10,17,18]^ Hence, TPM is a new drug for the treatment for seizures in neonates with HIE, but is currently used off-label.^[15]^ Our study aims to investigate the efficacy and safety of TPM for HIE which will help us have better understanding in TPM as treatment for HIE.

Although there are some randomized controlled trials (RCTs) or reviews studying effects on TPM for HIE, no comprehensive quantitative reviews of TPM for HIE have been performed. Therefore, it is significant to investigate the present studies of the efficacy and safety of TPM therapy for HIE.

## 2 Method

The systematic review will be developed in compliance with the Preferred Reporting Items for Systematic Reviews and Meta-Analyses (PRISMA) approach and reported adhering to the Preferred Reporting Items for Systematic Reviews and Meta-Analyses Protocols (PRISMA-P) 2015 statement. It has been registered on PROSPERO (ID: CRD42018117981)

### 2.1 Eligibility criteria

#### 2.1.1 Participants

All patients who are newborn babies with perinatal asphyxia developing to HIE, without limitation of gender, geographic area.

#### 2.1.2 Intervention

There will not be any group limitation that TPM is used in, which means that intervention group or control group using TPM will be included, but TPM should be compared with another therapy, such as other medicine, placebo, blank or TPM combining with other medicine.

#### 2.1.3 Study design

Randomized controlled trials.

#### 2.1.4 Comparison

The control group uses other treatment measures except TPM, such as memantine, phenytoin, midazolam or other drugs.

#### 2.1.5 Exclusion criteria

The studies that the patients did not be diagnosed as HIE, the diagnosis code is not clear, the patients diagnosed HIE complicating other diseases will be excluded. The articles will be excluded like editorials, literature reviews, case reports, letters, conference abstracts, and dissertations.

#### 2.1.6 Evaluated outcomes

*Primary outcomes*. Reduce seizure acticity.

*Secondary outcomes.* Reduction in mortality, severity of brain damage assessed with MRI, oxidative stress, as well as adverse events.

### 2.2 Electronic search

Four English databases, PubMed, the Cochrane Central Register of Controlled Trials (Cochrane Library), Embase, and Web of Science, and 4 Chinese databases, China National Knowledge Infrastructure (CNKI), Chinese Biomedical Literature Database (CBM), Wang Fang Database and Chinese Science and Technology Periodical database (VIP) will be searched comprehensively for TPM for HIE.

### 2.3 Other sources

To find out all the eligible articles, we will scan the conference proceeding, International Clinical Trials Registry Platform and Chinese Clinical Trials Registry in electronic or manual way.

### 2.4 Search strategy

The strategies will be developed by the medical subject headings and keywords. A strategy for PubMed in detail is shown in Table 1 and will be modified for the other databases.

**Table 1:**
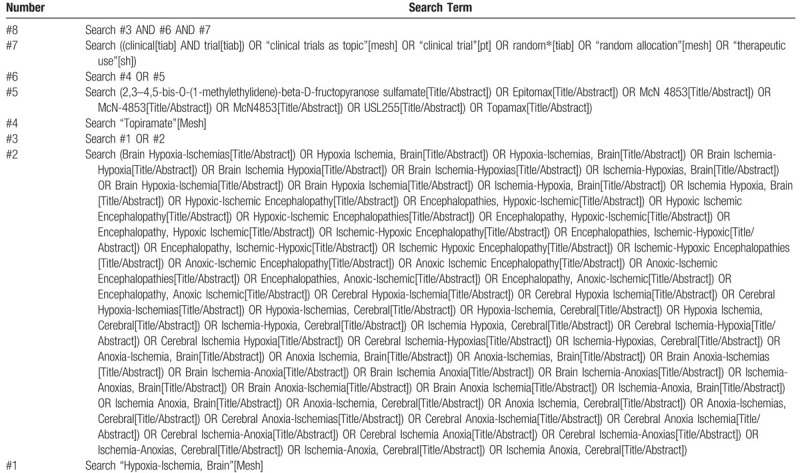
Search strategy for the PubMed database.

### 2.5 Study selection

**Figure 1:**
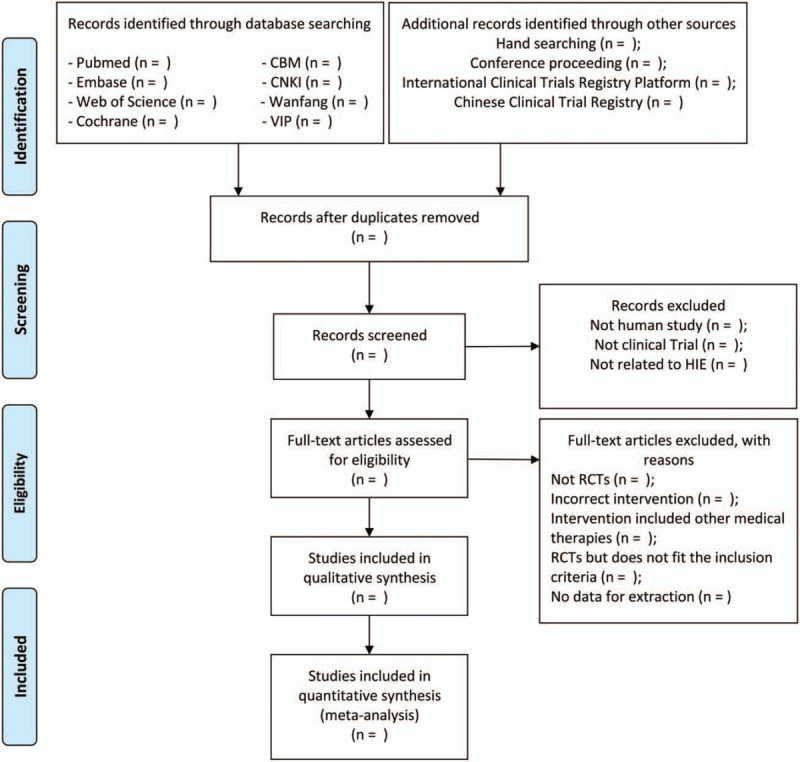
PRISMA flow chart of study selection process.

To find out all eligible articles, 2 experienced researchers will use the Endnote X9 to import the articles, then remove the repetitive or disqualified literature through screening full texts based on the inclusion and exclusion criteria. If there is any disagreement, all the researchers will discuss and solve it. If necessary, the third researcher will be consulted. The process of selecting the eligible literature is shown in a Preferred Reporting Items for Systematic Reviews and Meta-Analyses (PRISMA) flow diagram (Fig. 1).

### 2.6 Data collection and analysis

When finish identifying all the eligible literature, the other 2 researchers will independently use the pre-prepared extraction forms to make the data extraction according to the topic, first author, published journal, published date, and the details of the studies including information of the participants in the intervention and control group, treatments and comparisons, sample size, outcomes, adverse events, and etc. If disagreements appear, the third reviewer will solve them.

The Cochrane's tool recommended by Cochrane Handbook for Systematic Reviews of Interventions will be used by 2 trained reviewers to assess the risk of bias in the RCTs. The following items will be used to assess each primary study: random sequence generation, allocation concealment, blinding method for participants and personnel, blinding of outcome assessments, incomplete data, selective reports, and other biases. All the articles will be classified to 3 levels: low risk, unclear risk, or high risk.

Employing RevMan version5.3, we will conduct a meta-analysis. The categorical data will be presented as rare ratio (RR) for dichotomous outcomes, and continuous data will be computed as mean different (MD). Categorical and continuous variables will be expressed with 95% confidence intervals (CI) for analysis.

If the required data is unclear or not shown in the collected papers, the researchers will contact the first or corresponding authors via phone or email for the detailed information. If no respondence is received, we will analyze outcome using the available data and assess the possible influence the missing data might cause on the conclusion.

Heterogeneity will be evaluated by using *I*^2^. If *I*^2^ > 50%, we consider that the significant heterogeneity is exist, and detect the potential cause by performing a subgroup analysis and managing a descriptive statistical analysis for data synthesis. If *I*^2^ < 50%, the heterogeneity is regarded as low and the statistical heterogeneity will be searched via Chi-squared test.

If there is more than 10 pieces of included trials in the review, a visual asymmetry will be made on a funnel plot via Egger methods to detect the potential reporting biases or small-study effects.

### 2.7 Data synthesis

If there are appropriate results in meta-analysis, we will compute the data synthesis by using RevMan V5.3. The fixed effects model will be employed to perform the analysis if no obvious statistical heterogeneity among the studies is observed. On the contrary, if obvious clinical heterogeneity is found, the random effects model will be used to carry out the analysis. Meanwhile, subgroup or sensitivity analysis will be done by the researchers when the heterogeneity is found. If meta-analysis cannot be performed, only a descriptive analysis making a summary and explaining the characteristics and results will be made.

### 2.8 Subgroup analysis

Subgroup analysis will be conducted to explore the resources of the heterogeneity. We will consider all the factors followed: inconsistent patient characteristics, classifications of HIE, disease courses, different types of interventions, outcome measures, and other unpredictable factors.

### 2.9 Sensitivity analysis

If necessary, sensitivity analysis will be applied to investigate funnel plot asymmetry. Following methodological qualities, sample size and the effect of missing data, the sensitivity analysis will be carried out. After excluding the studies of high-risk bias and the outcomes, we will make the meta-analysis again and take it into comparison with the original one.

### 2.10 Quality of evidence

The quality of the results will be assessed by The Grading of recommendations Assessment Development and Evaluation (GRADE).

### 2.11 Ethics and dissemination

Ethical approval and the informed consent are not required for this research is not the clinical study and personal information is not involved. Peer-viewed publications and related conferences are in the dissemination plan. The evidence of this review make a contribution to summarize the results of TPM in treatment of HIE, from which doctors will derive a deeper knowledge of the efficacy and safety of TPM. Updating this study is helpful for informing and orienting clinical practice.

### 2.12 Patient and public involvement

Neither patients nor public are involved.

## 3 Discussion

TPM is known as an anti-epileptic drug used for some time. However, the drug has large amounts of side effects, only approved to utilize for over 2-year-old minors. Researches on TPM treating patients with HIE, especially neonates, are also limited for a long period of time since the drug was synthesized. In the past few years, its potential therapeutic effect on brain injury such as HIE has been also taken attention. Recent animal experiments and clinical studies indicated that short-term application of the drug might benefit the patients with HIE.^[19–21]^

Up to now, there is no published systematic review on TPM for the treatment of HIE. This study synthesized relevant data systematically to reflect the integrated efficacy of the trials. It showed that TPM had positive curative effect on HIE. However, our study did not involve analysis of adverse effects caused by TPM and long-term application of the drug. Due to indications of contemporary clinical practice of TPM, only a few trials are eligible after retrieved and filtered. More randomized controlled trials are required to enrich utilization of TPM.

## Author contributions

**Data curation:** Tengyu Chen, Zhaoping Zhang, Yingyue Hou, Wanli Xing.

**Methodology:** Yijun Chen, Yaying Xie, Ruilan Huang.

**Project administration:** Guoming Chen, Li Wei.

**Software:** Tengyu Chen, Peiyu Shi.

**Writing – original draft:** Guoming Chen, Yijun Chen, Yaying Xie, Ruilan Huang, Tengyu Chen, Peiyu Shi, Zhaoping Zhang, Yingyue Hou, Wanli Xing.

**Writing – review & editing:** Guoming Chen.

Li Wei orcid: 0000-0003-4682-0584.
